# Gastrogastric Intussusception 10 Years After Laparoscopic Gastric Greater Curvature Plication: a Case Report

**DOI:** 10.1007/s11695-024-07402-2

**Published:** 2024-08-01

**Authors:** Mohamed Sharaan, Mohamed M. Okba, Mohamed Ahmed El Badawy, Bart Torensma, Mohamed Hany

**Affiliations:** 1 Faculty of Medicine, Alexandria University, Alexandria, Egypt; 2 Leiden University Medical Center (LUMC), Leiden, The Netherlands; 3 Department of Surgery, Medical Research Institute, Alexandria University, 165 Horreya Avenue, Hadara, Alexandria, 21561 Egypt; 4 Madina Women’s Hospital, Alexandria University, Alexandria, Egypt

## Introduction

Metabolic bariatric surgery (MBS) is the leading long-term solution for obesity. The surge in MBS demand has led to innovations, including modernizing old techniques with minimally invasive methods. Gastric greater curve plication (LGCP) was first performed in 1976 and later adapted to a laparoscopic method in 2007. It served as a laparoscopic sleeve gastrectomy (LSG) alternative. This technique, involving the folding and suturing of the stomach’s greater curvature, offers reversibility and reduces the risk of leakage without removing stomach tissue. Despite LGCP’s intent to enhance the LSG procedure, recent studies show a consensus that LSG outperforms LGCP in perioperative safety, weight loss efficacy, and long-term complications like gastric obstruction, abscess formation, gastric prolapse, or release of the plicated wall [1–4].

## Purpose

This multimedia article presentsa case of gastrogastric intussusception 10 years after LGCP.

## Case Presentation

A 31-year-old woman with a history of LGCP in May 2013 presented with 2 years of frequent vomiting, colic, and regurgitation. In June 2023, the patient’s complaint was exacerbated, and she sought medical advice at our center. She was pale, with epigastric and right hypochondrial tenderness, but no abdominal masses. Labs showed mild anemia (hemoglobin 10.9 g/dL). The patient was diagnosed with hyperlipidemia through a laboratory test. She is not currently taking any medication for this condition. However, the internist has prescribed a lipid-lowering drug for her. The patient’s cholesterol level was measured to be 277 mg/dL, triglycerides at 155 mg/dL, HDL at 34 mg/dL, and LDL at 200 mg/dL. Additionally, the patient was suffering from hypertension, which was controlled with an antihypertensive drug from the beta-blocker class. She was also taking prokinetics and a proton pump inhibitor.

Her weight was 89 kg (body mass index (BMI) 33.7 kg/m^2^), down from a preoperative weight of 101 kg (preoperative BMI 39.4 kg/m^2^), achieving a lowest weight of 72 kg (BMI 28.1 kg/m^2^), which she maintained for 3 years before recurrence in weight. She tolerated fluids and a soft diet during vomiting episodes.

**Fig. 1:**
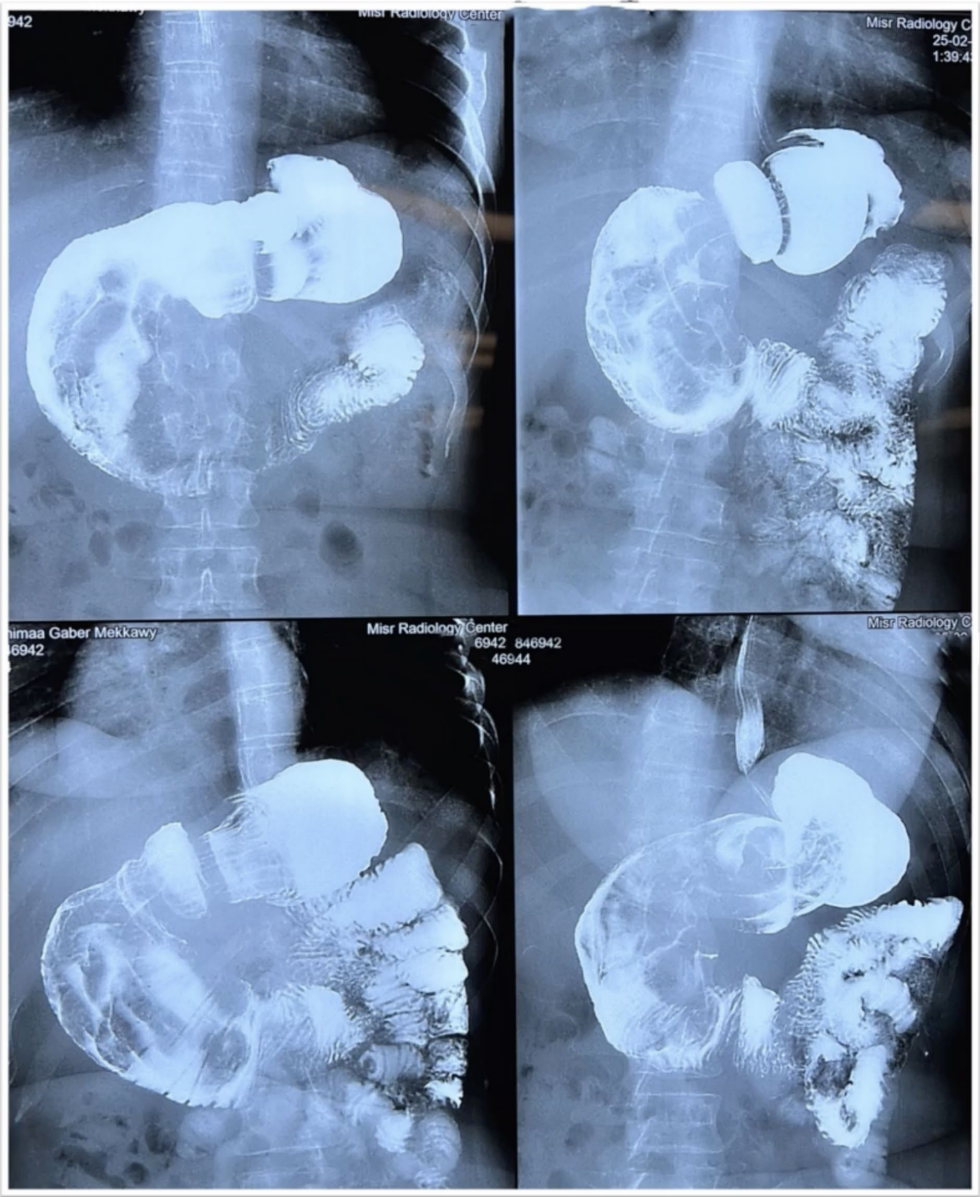
Digital barium meal

**Fig. 2:**
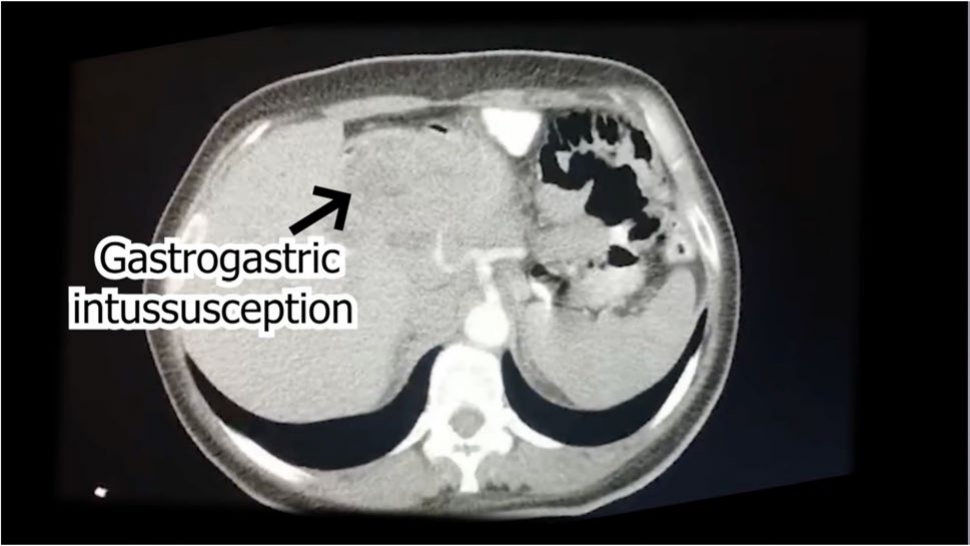
CT scans of gastrogastric intussusception

A digital barium meal showed the stomach’s pyloric part dilated, twisted, and rotated, with the duodenal cap pointing left and regular duodenum filling. Computed tomography (CT) imaging indicated stomach fundus and pylorus invagination into the duodenum, suggesting intussusception. Upper gastrointestinal (GI) endoscopy revealed difficulties entering the duodenum, with visible dilation and plication folds in the gastric pouch, but no hiatal hernia or reflux was observed (Figs. 1 and 2).

After discussing this with the patient, it was decided that laparoscopic exploration should proceed. The patient wanted to maintain or lose more weight, so we reviewed various options. Due to the nature of the surgery requiring resection, two options were presented: LSG or resectional bypass. We explained the potential drawbacks of each surgery, including the possibility of reflux. After thoroughly discussing these options, the patient ultimately preferred LSG. She had concerns about the potential challenges of reversing a bypass in the future, as well as fears of encountering nutritional deficiencies associated with different types of bypasses.

### Conversion Operation

Laparoscopic exploration successfully reduced intussusception with instrument assistance, a finding corroborated by intraoperative endoscopy, which identified a gastric fold mimicking a polyp. Subsequently, the plicated stomach wall was unfolded, and LSG was completed using a 40 Fr calibration tube, with a tube drain placed at the end of the surgery. Examination of the gross specimen revealed no abnormal masses, and histopathology identified chronic gastritis without dysplasia. The patient experienced a smooth postoperative recovery, being discharged on the second day following drain removal (Appendix 1, a full Step-by-Step Description of the operation).

### Postoperative Course

The surgery occurred in July 2023, and the postoperative recovery was uneventful. The patient was discharged on the second postoperative day after removing the drain. She had no difficulties tolerating oral fluids. Follow-up appointments with the surgeon were scheduled for 3 weeks, 3 months, and 6 months after the surgery, with an additional one-year follow-up appointment. Additionally, she has monthly appointments with a nutritionist and three-month appointments with a psychiatrist as part of our center's routine during the first year. She is taking MBS multivitamins (Elan, Believe, for sleeve gastrectomy, the Netherlands), Ferro (Elan, the Netherlands), and proton pump inhibitors as necessary.

### Follow-up

In May 2024, 10 months later, the patient had lost 24 kg, bringing her current weight to 65 kg. She reported no colic but occasional episodes of vomiting due to rapid eating habits. Hypertension was resolved, and hyperlipidemia showed improvement. The 6-month follow-up revealed improvements in previously abnormal laboratory tests. The blood picture indicated improvements, with a hemoglobin level of 11.7 g/dL, ferritin at 55 ng/mL, iron at 17 micromol/L, High-density lipoprotein (HDL) cholesterol at 45.3 mg/dL, total cholesterol at 168.5 mg/dL, triglycerides at 101.7 mg/dL, low-density lipoprotein (LDL) at 102.8 mg/dL, and a cholesterol/HDL risk ratio of 3.72.

## Discussion

This was a report of a rare case of gastrogastric intussusception after LGCP. Recently, an article about gastroduodenal intussusception was published, describing a case managed through laparotomy due to acute abdomen and leucocytosis. This incident occurred five years after LGCP [5]. Another patient required reoperation for gastric obstruction 14 months post-LGCP due to the prolapse of the plicated gastric fundus into the duodenum [6]. Almulaifi and Mohammad reported a rare case of obstructive jaundice six months after LGCP caused by gastric fold herniation into the duodenum [7]. Additionally, a case was reported following gastric bypass where the gastric remnant of a previously plicated stomach protruded into the duodenum [8]. It is essential to differentiate between prolapse and intussusception. Prolapse refers to the gastric tissue protruding between stitches, which can lead to missed events during a search due to different terms such as prolapse, protrusion, and herniation. These terms provide different perceptions regarding the nomenclature of rare complications like intussusception. We believe our case is of a chronic nature, occurring ten years after LGCP, which is significantly later than previously reported cases. Furthermore, our case is not presented as a video presentation with management.

Over the last decade, numerous studies have compared LSG and LGCP. Two systematic reviews (SRs) conducted in 2018 provide foundational insights. Perivoliotis et al. performed a systematic review and meta-analysis of 12 studies, concluding that LSG demonstrated a lower overall complication rate and higher weight loss than LGCP. They emphasized the need for further randomized controlled trials (RCTs) of higher methodological quality and larger sample sizes to validate these findings. Similarly, Barrichello et al. published an SR and meta-analysis of 8 studies, which found that LSG resulted in better weight loss, improved satiety, fewer postoperative symptoms, and better diabetes remission compared to LGCP [2, 3]. The most recent SR by Li et al. in 2021 updated the analysis to include 18 studies, encompassing 1329 patients (705 LSG and 624 LGCP), including 5 RCTs. Major complications evaluated included bleeding, stenosis, leak, thromboembolism, and mortality, while secondary complications comprised nausea, vomiting, GERD, wound infection, port-site hernia, sialorrhea, and abdominal pain [9]. Contrary to expectations, this meta-analysis did not show any advantage of LGCP in reducing complications. Bleeding, stenosis, and mortality rates were comparable between the two procedures. Notably, only 12 cases of leakage were reported across all 18 studies, with no significant difference between LSG and LGCP, highlighting that LGCP does not reduce the risk of leakage—a serious complication of LSG. Most of the included studies reported only short- or mid-term results, with long-term follow-up (4 years or more) lacking, and no reports on revision surgery were included as this was not the focus of the SRs. Furthermore, only 5 of the 18 included studies were RCTs, which may have introduced selection and detection biases. The SR by Li et al. excluded case reports and series, potentially underestimating the incidence of rare complications after LGCP [9]. Additional studies provide further context. In 2017, Zerrweck et al. reported a high failure rate for LGCP, with an increased number of symptomatic patients. Revisional surgery proved safe and effective, with revision to LSG being faster and involving shorter hospital stays, while revision to Roux-en-Y gastric bypass (RYGB) showed better excess weight loss (%EWL) at 18 months [10]. A 2021 study by Ibrahim et al. compared LSG and LGCP over at least 5 years, involving 163 patients. The primary reasons for revision were gastroesophageal reflux disease (GERD) or bile reflux in the LSG group and weight gain in the LGCP group. LSG was superior in improving associated medical problem. Endoscopic findings frequently showed GERD (grade A or B) in the LSG group and unfolding of part or the entire stomach in the LGCP group. Early minor postoperative complications were significantly less frequent in the LSG group. Overall, LGCP had a higher rate of complications, less durable weight loss, a higher rate of revision surgery, and a greater cost burden on the healthcare system compared to LSG [4]. These findings underscore the importance of informing patients about the experimental nature and potential risks of LGCP. It is essential to communicate that LGCP is not yet an accredited type of surgery and remains an experimental practice, as highlighted in ethical considerations for surgery in an IFSO Position Statement [11]. Routine endoscopic surveillance is recommended to monitor for long-term complications. An International Federation for the Surgery of obesity and metabolic disorders (IFSO) Position Statement advocates for regular endoscopic evaluations to detect complications specific to certain types of surgery, including LGCP. Adherence to these guidelines is crucial for the early detection and management of potential issues [12]. In conclusion, our study emphasizes the need for awareness and proactive management of rare complications associated with LGCP. By following recommended guidelines and maintaining open communication with patients about the experimental nature and risks of the procedure, we can improve patient outcomes and advance surgical practice.

## Directions for Future Research

Future research should focus on long-term follow-up studies to evaluate the durability of weight loss and the persistence of complications over an extended period. Studies with follow-up periods of 5 years or more would provide valuable insights into the sustainability of LGCP outcomes. Comparative studies between LGCP and other bariatric procedures, such as LSG and RYGB, should be conducted to evaluate differences in weight loss, complication rates, and resolution of associated medical problems. These studies should include large sample sizes and be highly methodological to ensure robust findings. More RCTs are needed to provide high-level evidence on the effectiveness and safety of LGCP compared to other bariatric procedures. These trials should minimize bias and include diverse populations to enhance the generalizability of the results.

Research should include assessments of patient quality of life and satisfaction post-surgery. Evaluating psychosocial outcomes and the impact of surgery on daily functioning can provide a comprehensive understanding of LGCP's benefits and drawbacks. Studies examining the outcomes of revision surgeries following LGCP are necessary. These should assess the safety, efficacy, and patient satisfaction of converting LGCP to other bariatric procedures, such as LSG or RYGB, particularly in cases of weight regain or complications. Research should investigate the underlying mechanisms of complications associated with LGCP, such as intussusception and bleeding from sutures. Understanding these mechanisms can lead to improved surgical techniques and preventive measures. Future studies should include cost-effectiveness analyses to compare the long-term economic impact of LGCP with other bariatric procedures. This would help inform decisions about the most efficient use of healthcare resources. Research should explore the ethical implications of using LGCP as an experimental procedure. This includes evaluating informed consent processes and ensuring that patients are fully aware of the risks and benefits associated with LGCP.

## Conclusion

Gastrogastric intussusception is a rare complication that has been reported after LGCP. However, the presentation of this complication after long-term follow-up has not been reported. Gastric folds may play a significant role in triggering its occurrence. As this complication persists chronically and attempts to reduce it endoscopically have been unsuccessful, it is advisable to consider laparoscopic surgical reduction. This procedure includes unfolding and converting it to an alternative technique.

## Supplementary Information

Below is the link to the electronic supplementary material.

Supplementary file1 (MP4 168262 KB)

## Data Availability

Data available with the corresponding author.

## Appendix 1 Step-by-Step Description

After discussing with the patient, it was decided to proceed with laparoscopic exploration. The patient wanted to maintain or lose more weight, so we reviewed various options. Due to the nature of the surgery requiring resection, two options were presented: sleeve gastrectomy (LSG) or resectional bypass. We explained the potential drawbacks of each surgery, including the possibility of reflux. After thoroughly discussing these options, the patient ultimately preferred LSG. She had concerns about the potential challenges of reversing a bypass in the future, as well as fears of encountering nutritional deficiencies associated with different types of bypasses.

**The following steps were taken during the operation:**

- Initially, we located the site of the intussusception.
- Using a 5 mm laparoscopic instrument, we gently moved it from the pylorus towards the patient's left and upwards, successfully reducing the intussusception.
- Intraoperative endoscopy was performed to confirm the reduction. In the “retroflex view,” we observed a plicated fold in the fundus area, and the endoscope could pass through the first part of the duodenum.
- We began adhesiolysis using a Ligasure (Medtronic, USA) to free the plicated stomach completely.
- With the assistance of the Ligasure and scissors, we unfolded the plicated stomach.
- After unfolding, a 40 Fr calibration tube was inserted through the stomach.
- We started stapling the stomach 6 cm from the pylorus using a Tristapler black reload (Medtronic, USA) for the first firing, followed by purple reloads until completion, 1 cm lateral to the angle of His.
- Nonabsorbable 3/0 Prolene (Ethicon Inc, Johnson & Johnson, USA) sutures were used to reinforce the upper part of the staple line from the last two firings.
- After widening the left 12 mm port, the stomach was extracted, and the port was closed using a fascial closure needle and Vicryl 2/0 (Ethicon Inc, Johnson & Johnson, USA) to approximate the edges.
- An 18 Fr tube drain was inserted at the surgical bed.

We examined the specimen closely and found no abnormal masses or evident residual folding. The histopathological examination indicated chronic gastritis without any detected dysplasia.
