# Correction to: Gastrogastric Intussusception 10 Years After Laparoscopic Gastric Greater Curvature Plication: a Case Report

**DOI:** 10.1007/s11695-024-07448-2

**Published:** 2024-10-09

**Authors:** Mohamed Sharaan, Mohamed M. Okba, Mohamed Ahmed El Badawy, Bart Torensma, Mohamed Hany

**Affiliations:** 1 Faculty of Medicine, Alexandria University, Alexandria, Egypt; 2 Leiden University Medical Center (LUMC), Leiden, The Netherlands; 3 Department of Surgery, Medical Research Institute, Alexandria University, 165 Horreya Avenue, Hadara, 21561 Alexandria Egypt; 4 Madina Women’s Hospital, Alexandria University, Alexandria, Egypt

**Correction to: Obesity Surgery**

10.1007/s11695-024-07402-2

The original article has been corrected to delete the abstract.

